# From Hydrogen Bonding to Hydrophobic Control: The Shifting Solvation Mechanism of Ibuprofen in Deep Eutectic Solvents

**DOI:** 10.1021/acs.jpcb.5c08530

**Published:** 2026-06-20

**Authors:** Vinicius Piccoli, Agílio Pádua, Leandro Martínez

**Affiliations:** † Institute of Chemistry and Center for Computing in Engineering & Science, 28132 Universidade Estadual de Campinas (UNICAMP), Campinas 13083-872, SP, Brazil; ‡ Chemistry Laboratory, 26911 École Normale Supérieure (ENS) de Lyon, 46, allée d’Italie, 69364 Lyon Cedex 07, France

## Abstract

This study investigates the solvation of ibuprofen in deep eutectic solvents (DESs) composed of the hydrogen bond acceptor (HBA) betaine (BET) and hydrogen bond donors (HBDs) ethylene glycol (EG), propylene glycol (PG), or 1,2-butanediol (BD). Molecular dynamics simulations were performed using the Drude polarizable model with a custom-implemented Tang-Toennies damping function designed to resolve short-range polarization artifacts. The structure and thermodynamics of solvation were characterized with minimum-distance distribution functions (MDDFs) and the Kirkwood–Buff (KB) theory of solutions. Betaine forms strong hydrogen bonds with the carboxylic group of ibuprofen, whereas alcohols, especially EG, are largely excluded from the first solvation shell. Decomposition of solvent MDDFs reveals that hydrophobic groups from ibuprofen promote the accumulation of both betaine and HBDs, with the preferential solvation shifting from betaine to the HBD as the alkyl chain increases. Betaine preferentially binds ibuprofen with greater preference in the BET–EG system, while in the BET–BD system, the hydrogen-bond donor is preferentially accumulated around the solute. Beyond characterizing these specific interactions, this work demonstrates the application and usefulness of MDDF-based KB analysis in anhydrous, highly viscous media, extending its application from traditional aqueous protein systems to the complex, glass-like dynamics of deep eutectic environments.

## Graphical abstract

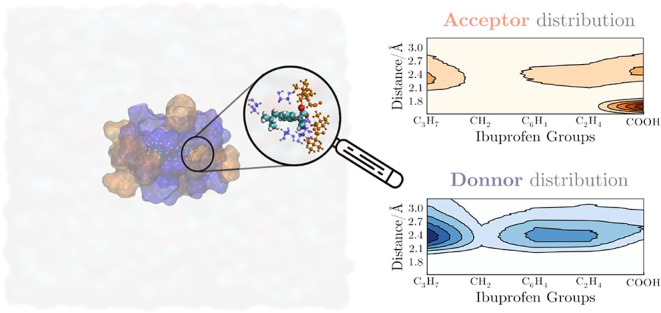

## Introduction

Deep eutectic solvents (DESs) are most broadly defined as mixtures where the melting point, including the eutectic point, is significantly lower than that predicted for an ideal mixture of the components.^1^ This phenomenon is obtained by combining hydrogen bond donor (HBD) and hydrogen bond acceptor (HBA) species which might be solid at room temperature, but when combined in the appropriate molar ratio, form a liquid phase.^2^ The versatility of these systems stems from a vast array of potential cationic, anionic, or zwitterionic species that can be used for their formulation. For instance, betaine is a commonly used zwitterionic molecule used to obtain DESs.^2^ With betaine, as well as with ammonium salts, the interactions between the HBA (such as complementary halide anion in a salt, or carboxylate group of a zwitterion) and the HBD disrupt the crystalline lattices. Early theories emphasized the role of charge delocalization in this disruption, but recent studies suggest that the deep eutectic behavior arises from a complex balance between enthalpic and entropic effects on the solution.^2–6^

The compositional flexibility of DESs exceeds that of ionic liquids (ILs), although research into ILs and IL–water mixtures has provided a foundational framework for understanding solvation, preferential interactions, and biomolecule stabilization.^7–12^ Like ILs, DESs have gained attention in catalysis,^13^ nanotechnology,^14^ biomass processing,^15^ separations,^16^ and materials synthesis.^17^ In particular, the application of DESs in pharmaceutical science has drawn significant interest due to their capacity to enhance drug solubility,^18^ stabilize proteins,^19^ and serve as tunable drug delivery media.^13,20^

For example, a challenge in the pharmaceutical development and clinical application of nonsteroidal anti-inflammatory drugs (NSAIDs), like ibuprofen, is their low aqueous solubility.^21^ While many of these compounds are marketed in their salt form, such as diclofenac sodium, diclofenac potassium, and ibuprofen sodium, salt formation is not a universally applicable strategy. For example, zaltoprofen is particularly difficult to ionize because of its weakly acidic nature, rendering traditional salt-based solubilization ineffective.^21,22^ Furthermore, attempting ionization can lead to unwanted polymorphic transformations, which pose a significant risk to the drug’s stability and therapeutic activity.^23^ Consequently, it is important to develop new approaches to improve the solubility and overall physicochemical properties of these poorly soluble drugs.^24,25^

Biocompatible DESs are increasingly utilized as functional excipients to enhance the solubility,^26^ bioavailability, and dissolution rates of poorly water-soluble drugs.^27,28^ The pharmaceutical viability of these systems is supported by their favorable toxicological profiles, with betaine-based DESs, in particular, offering superior ecocompatibility and lower cytotoxicity compared to traditional choline-chloride-based counterparts.^29,30^ For example, the solubility and topical delivery of dapsone, an antimicrobial and anti-inflammatory agent primarily used to treat leprosy, were dramatically enhanced by choline-chloride-based DESs. While dapsone exhibits a small aqueous solubility of 380 mg/L, a system pairing choline chloride with propylene glycol reached a drug concentration of 500 mg/mL, representing a significant increase in its solubilization profile.^31^ Beyond simple solubilization, pure deep eutectic solvents are being developed as primary delivery vehicles for specialized treatments, such as burn wound care.^32,33^ These formulations function as concentrated ointments that leverage the inherent antimicrobial properties of the eutectic components to reduce infection risks and minimize scarring during the healing process.^32,33^

A molecular-level understanding of how DESs interact with drug molecules remains limited, particularly due to the complexity introduced by strong hydrogen bonding networks, viscosity, and microscopic heterogeneity of the DES medium.^34–36^ Computational methods offer a pathway to unravel these complexities by providing atomic-scale insights that complement experimental approaches. Among these methods, classical molecular dynamics (MD) simulations have been instrumental in capturing solute–solvent interactions, solvation structures, and thermodynamic properties. Traditional fixed-charge force fields often fail to capture essential features of DESs, notably polarization effects. In highly polar or ionic systems such as DESs, the local electronic environment can induce significant fluctuations in atomic dipole moments, which fixed-charge models cannot accommodate. With these shortcomings, fixed-charge models have failed to reproduce thermodynamic and transport properties of ionic media simultaneously.^37,38^

In this work, we employ polarizable molecular dynamics simulations to investigate the solvation of ibuprofen in three DESs composed of betaine (HBA) and different HBDs: ethylene glycol (EG), propylene glycol (PG), and 1,2-butanediol (BD). Ibuprofen was modeled in its neutral, protonated form (COOH), which allows it to act as a hydrogen bond donor. This choice is consistent with the chemical environment of these DESs, where the absence of strong bases and the zwitterionic nature of betaine, simulated here with its carboxylate (COO^–^) and ammonium (N­(CH_3_)_3_^+^) groups, favor the maintenance of the protonated solute. The glycols, being neutral molecules with weak acidic hydroxyl groups, function as secondary hydrogen bond donors. Under these conditions, the primary interaction occurs between the carboxyl group of ibuprofen and the carboxylate site of betaine. Using a combination of minimum-distance distribution functions (MDDFs), Kirkwood–Buff (KB) integrals, and preferential solvation parameters, we examine the structural and thermodynamic features governing these interactions. We demonstrate an inversion in preferential solvation driven by the increasing hydrophobicity of the glycol; specifically, ibuprofen solvation can be tuned by the competition between the hydrogen-bonding capacity of the HBA and the hydrophobic character of the HBD.

## Methods and Theory

### Computational Details

This section details the computational methodology employed to investigate the solvation of ibuprofen in three distinct deep eutectic solvents (DESs). These DESs were formed by combining the zwitterionic betaine (BET) as the hydrogen bond acceptor (HBA) with three different hydrogen bond donors (HBDs): ethylene glycol (EG), propylene glycol (PG), and 1,2-butanediol (BD). The selection of these simple diols, commonly found in DES formulations, allowed for a systematic investigation of the influence of the HBD alkyl chain length on ibuprofen solvation. Figure 1 illustrates the chemical structures of the molecules studied. The systematic variation in the alkyl side chain length of the HBDs (EG, PG, and BD) was designed to investigate the potential impact of dispersive-like interactions between the HBDs and the ibuprofen solute on the solvation process. The DES mixtures were prepared in a 1:3 HBA:HBD molar ratio. This stoichiometry is consistent with previous experimental studies for the BET–EG and BET–PG systems;^39^ for the BET–BD system, the same 1:3 ratio was adopted to maintain consistency across the series and allow for a direct comparison of the HBD alkyl chain effect.

**Figure 1:**
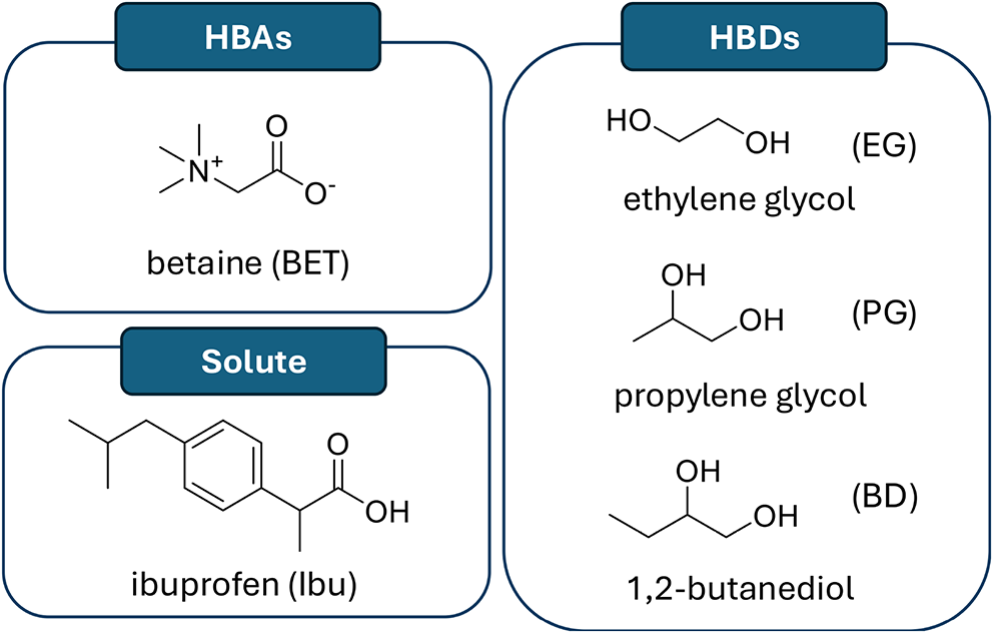
Chemical structures of the compounds used to simulate the solvation of ibuprofen in deep eutectic solvents.

The molecular dynamics simulations employed the polarizable CL&Pol force field.^34,40,41^ This force field is based on the OPLS-AA parameter set for alcohols and other molecules and on the CL&P fixed-charge force field for ionic liquids.^42^ While CL&Pol parameters were available for ibuprofen, EG, PG, and BD, the partial charge on the nitrogen atom of the BET zwitterion was calculated separately (the procedure and charges of the BET atoms are detailed in Table S3 of the Supporting Information). The initial simulation boxes, each containing one ibuprofen molecule at infinite dilution in the respective 1:3 HBA:HBD DES mixtures, were constructed using Packmol.^43,44^ The force field topology and parameter files required for the simulations were then generated using fftool.^45^ Three distinct DES systems were investigated: BET:EG, BET:PG, and BET:BD.

Here we opt to use a physics-based polarizable model (CL&Pol) rather than a traditional fixed-charge or an empirically scaled-charge framework to capture the true multicomponent properties of zwitterionic deep eutectic solvents. Betaine features a strongly basic carboxylate (COO^–^) group and a bulky trimethylammonium core, generating intense local electric fields that fluctuate dramatically across spatial and temporal scales.^34,37^ The physical implications of accounting for explicit electronic polarizability over additive approximations map directly onto the structural, dynamical, and thermodynamic properties of the condensed phase.

In traditional fixed-charge force fields, atomic partial charges are statically enhanced to mimic an average condensed-phase polarization state. In highly polar or ionic mixtures, this rigid approximation fails to accommodate the local environment’s dynamic asymmetry.^35,40^ This structural rigidity induces severe local overcoordination and artificial, short-range ion aggregation, which manifests as unphysically sharp, displaced nearest-neighbor coordination peaks. For DES systems specifically, this overstructuring has been shown to produce structural artifacts inconsistent with experimental neutron diffraction data, including artificial ion clustering and overstructured hydrogen-bond networks that are eliminated only when explicit polarization is included.^46,47^ In multicomponent DESs, this structural artifact can distort the hydrogen-bonding equilibrium between the zwitterion and the weakly acidic polyol hydroxyl groups, frequently inducing unphysical local phase separation or microheterogeneity.^34^

While polarizable simulations incur an extra computational cost per integration step, nonpolarizable models can, paradoxically, aggravate the phase-space sampling bottleneck by trapping the system in unphysically frozen states. A comprehensive review of polarizable force fields for ionic media has established that fixed-charge models with full integer charges yield self-diffusion coefficients, conductivities, and viscosities approximately 2 orders of magnitude off from experimental values, a systematic failure that is directly corrected when explicit polarization is introduced, confirming that the slow-dynamics problem is an artifact of the static charge approximation rather than a physical property of the system.^48^ Because fixed-charge frameworks overstabilize electrostatic networks through static charge definitions, their simulated transport properties degrade severely, yielding unphysically elevated bulk viscosities (η) and underestimated self-diffusion coefficients (*D*) by orders of magnitude.^49,50^ This failure is particularly acute in highly viscous DES media: nonpolarizable MD simulations of ionic systems produce dynamics nearly 1 order of magnitude too slow compared to experiment, and introducing Drude oscillator polarizability restores diffusion coefficients and correlation times to experimentally consistent values.^49^ By dynamically adapting atomic charge distributions, explicit polarization introduces an electronic screening layer that dampens the strong intermolecular Coulombic drag. This effectively lubricates the liquid matrix, accelerating translational and reorientational dynamics.^47^ Consequently, a polarizable framework provides localized relaxation and correlation times that are fundamentally more realistic, preventing the artificial structural glassiness that traps fixed-charge simulations in local free-energy minima.^49,50^

From a thermodynamic perspective, empirical alternatives such as uniform charge-scaling (typically reducing ionic charges to ±0.8e) systematically distort the system’s phase diagram and free-energy surfaces.^48^ Charge-scaling compromises the volumetric representation, underpredicting liquid densities by up to 5% and severely degrading the multibody cooperative energetics essential for hydrogen-bonded systems.^40,51^ Crucially, a scaled-charge model cannot properly reproduce the macroscopic dielectric response (ε) of a zwitterion-polyol matrix, which relies heavily on fluctuating molecular dipole moments.^51^ Explicit polarization models can accurately capture these fluctuating dipoles, which vary profoundly depending on the immediate local coordination environment, thereby yielding accurate solvation free energies, correct enthalpies of mixing, and a true physical representation of the solvent screening required to dissolve polar drug molecules.^35,52^ Therefore, the specific structural conclusions of this manuscript, namely the COOH···COO^–^ hydrogen-bond geometry between ibuprofen and betaine, and the preferential solvation inversion driven by HBD hydrophobicity, are not artifacts of static charge overestimation but reflect the genuine physical behavior of the system as captured by the polarizable model.

The systems were built in cubic boxes, each initialized with 85 Å of side length. To represent the experimental conditions, the number of molecules for each system was determined based on the experimentally measured densities of the DESs. This estimation of system composition was performed using the System Setup module of the MolSimToolkit.jl package, v1.29.0. The resulting final box dimensions (after NPT equilibration), the amount of each molecular component within the simulations, and the postequilibration concentrations are detailed in Table S1, in the Supporting Information.

The MD simulations were performed using the OpenMM toolkit,^53^ benefiting from GPU-accelerated hardware to enhance computational efficiency. Electronic polarization was explicitly accounted for through the Drude induced dipole model.^54,55^ The nonbonded interactions were truncated at a cutoff distance of 12 Å. Long-range electrostatic interactions were computed using the particle–particle particle-mesh (PPPM) method.^56^ Bond lengths involving hydrogen atoms were constrained using the SHAKE algorithm.^57,58^ Considering the high viscosity of deep eutectic solvents, all simulations were performed at a temperature of 353 K, maintained by the Drude-Nose-Hoover thermostat,^59,60^ coupling atomic and Drude particle degrees of freedom with damping frequencies of 5 and 20 ps^–1^, respectively, and under constant pressure of 1 bar using the Monte Carlo barostat.^61^ The Drude degrees of freedom (relative motion of Drude particles with respect to their atoms), representing electronic polarization, were thermostated at a low temperature of 1 K, following the cold Drude dipole approach.^62^ The temperature at 353 K allows the system to display faster relaxation and, for practical applications the compounds do not undergo thermal decomposition. For example, for many different betaine-based DESs, the decomposition temperature ranges from 106 to 233 celsius degrees.^2^

Ten (10) replicas, initiated from independent random solvent distributions generated by Packmol, were used to sample the solvation structure in each system. The MD protocol started with the system initially subjected to 10,000 steps of energy minimization to eliminate unfavorable contacts. After minimization, the systems underwent a 5 ns equilibration phase in the NPT ensemble at 353 K and 1 bar, employing a 1 fs time step. The final configurations from this phase were used as the starting point for production simulations. Production runs were then performed for 30 ns under the same NPT conditions, using the same integrator and barostat, and with a 2 fs time step, to ensure consistency in thermodynamic control throughout the simulations.

Other widely used molecular dynamics packages, such as LAMMPS,^63^ address the challenges associated with short-range charge-induced dipole interactions, often referred to as the ’polarization catastrophe’,^62^ through the implementation of Tang-Toennies-like damping functions. These functions provide a way to smoothly attenuate the electrostatic interaction at short distances, at which the point-charge approximation is no longer valid. Taking advantage of the flexibility offered by the OpenMM toolkit, we implemented, as described in the Supporting Information, a similar charge-dipole damping function, analogous to the Tang-Toennies approach, up to the fourth order of the series expansion.^37,64^ The PDBTools.jl^65^ package was used for structure handling and manipulation. Graphics were generated with Plots.jl,^66^ and DataFrames.jl^67^ was used for data handling. Distribution functions, coordination numbers, and hydrogen-bond analysis use the CellListMap.jl^68^ library for fast pairwise distance evaluations.

### Solvation Analysis

MDDFs describe the distribution of the shortest distance between solute and solvent atoms.^69^ They are particularly useful for representing interactions in molecules with complex shapes, as they inherently account for the molecular structure of both solute and solvent.^10,70^ The ComplexMixtures.jl package^71^ (https://m3g.github.io/ComplexMixtures.jl) was used to compute MDDFs,^70^ their decomposition into contributions from specific residue/chemical group types, density maps, Kirkwood-Buff (KB) integrals, and associated preferential interaction parameters.^10,72,73^ The solvent density at each distance was obtained from histograms of the average number of minimum distances, using 0.1 Å bins.

Figure 2 illustrates the convenience of using MDDFs over radial distribution functions (RDFs), in the present study. As sketched in panel 2A, ibuprofen is an anisotropic molecule with about 12 Å maximum length. The MDDF (*g*_*md*_ *(r)*) of betaine relative to ibuprofen, shown in the first plot of Figure 2A, indicates that there are two important solute–solvent interactions: one associated with hydrogen-bonding distances, at ∼1.9 Å, and nonspecific interactions, peaking at ∼3.5 Å. Decomposition of the MDDF into the contributions of the betaine atoms allows the precise association of these interactions with polar oxygen atoms and aliphatic hydrogens, respectively. On the other hand, the standard radial distribution, computed from an oxygen atom of betaine to center of mass of ibuprofen, shown in the second plot of Figure 2A, does not provide indications of hydrogen-bonding or interactions at 3.5 Å. The peak distances are noninformative and far from the actual distances of the molecular interactions because of the volume and shape of the molecules involved. To visualize the formation of the hydrogen bond, we need to compute specifically the RDF of the oxygen of betaine to the polar hydrogen of ibuprofen, as shown in the third plot of Figure 2A. The peak height differences are associated with the different reference volumes of each distribution.

**Figure 2:**
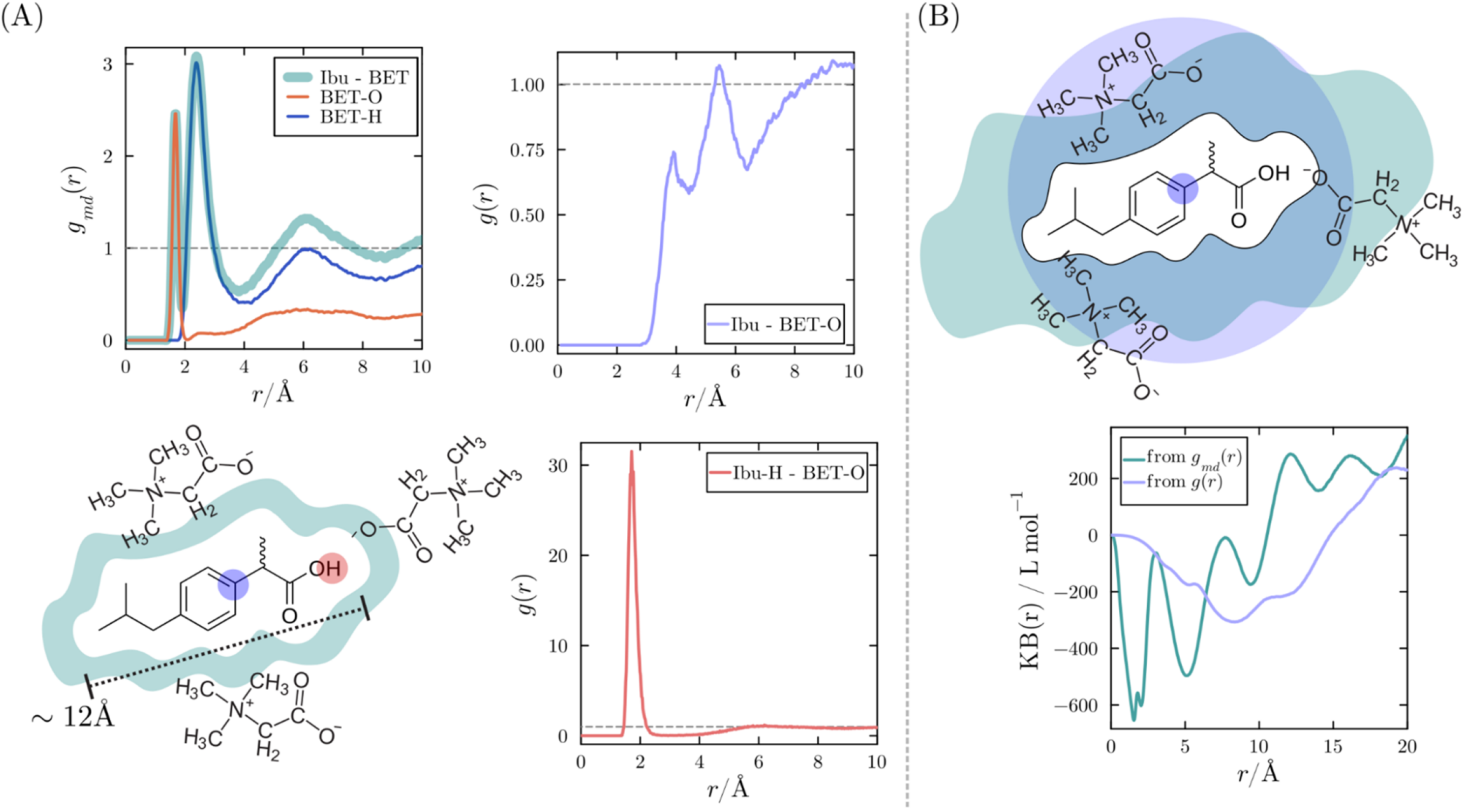
Minimum distance distribution functions vs radial distributions. (A) the molecular sketch illustrates the shape of the regions considered for the computation of MDDFs and RDFs, which are shown in the plots. MDDFs display peaks clearly associated with the solvent shell, while the RDF interpretation needs further manual inspection and recomputing for the characterization of specific interactions. (B) KB integrals computed from MDDFs or RDFs must converge to the same values, but the distances required for the proper convergence of RDFs are effectively larger because of the anisotropic shape of the solute.

As shown in Figure 2A, the center-of-mass RDF definition is clearly noninformative for probing local coordination due to the molecular envelope of the elongated solute. When substituting this descriptor with a site-specific, atom–atom RDF targeting the primary donor–acceptor pair (O_BET_···H_Ibu_), the distribution successfully captures the hydrogen-bonding peak at ∼1.9 Å with excellent clarity. For mixtures of moderate chemical complexity, site-specific RDFs are entirely appropriate and capture the identical local coordination features revealed by the MDDF analysis. The variation in their normalized peak heights is strictly a geometric consequence of their differing reference volumes. Consequently, the advantage of the MDDF-based framework is not an intrinsic superiority in capturing localized binding, but rather an operational convenience; its group-based decomposition provides the exact same multisite qualitative details under a unified, highly automated framework, providing equivalent multisite structural information within a single, automated computational framework.

The use of MDDFs, along with their straightforward decomposition into solute and solvent group contributions, is suitable for a comprehensive analysis of solvation structures, especially considering the complexity of DESs. The KB integrals can be computed from the counts of minimum distances between solute (*u*) and solvent (any other species, *c*) atoms for each distance *r* (*n*_*c*_(*r*)). The cumulative number of sites is obtained in the actual simulated system and in a reference state consisting of a noninteracting mixture with the same species and with the bulk density of the solvent (*n*_c_^*^(*r*))^70^

1 $$G_{\mathrm{uc}}=\frac{1}{\rho_{c}}\int_{0}^{\infty }\left[n_{c}\left(r\right)-n_{c}^{*}\left(r\right)\right]S\left(r\right)\mathrm{dr}$$

where *S*(*r*) is the surface area element at a distance *r* associated with the minimum distance from the solute. eq 1 reduces to the most typical radial distribution function when *n*_c_^*^(*r*) = ρ_*c*_ and *S*(*r*) = 4*πr* ^2^. When the integral of eq 1 is computed up to a finite distance *R*, it reduces to

2 $$G_{\mathrm{uc}}\left(R\right)\approx \frac{1}{\rho_{c}}\left[N_{\mathrm{uc}}\left(R\right)-N_{\mathrm{uc}}^{*}\left(R\right)\right]$$

where *N*_*uc*_(*R*) is the number of solute–solvent minimum distances is smaller than *R* in the solution, and *N*_uc_^*^(*R*) the MD count in the absence of solute–solvent interactions. *G*_*uc*_(*R*) converges when *R* is large enough such that the presence of the solute does not affect the distribution of the solvent molecules, i.e., when *n*_*c*_(*r*) ≈ *n*_c_^*^(*r*). The volume defined by the distance to the solute used to proclaim convergence is commonly referred to as the “solute domain”. Therefore, the KB integrals are computed from the fluctuations in the number of solvent molecules within the solute domain.

KB integrals provide a net measure of the accumulation of depletion of the components of the solution relative to the solute. Distribution functions provide a picture of the local density fluctuations of the solvent, but it is the integral of these fluctuations in the volume of the solution affected by the presence of the solute that provides a quantitative measure of the affinities of the solute with the solvent components. Importantly, this net solute–solvent affinity is what causes the observed variations in density, osmotic pressure, etc., connecting the microscopic distribution of the solution with macroscopic measurable quantities. As such, KB integrals must be independent of the microscopic representation of the density distribution (given a proper reference state). RDFs computed with different reference centers, spatial density distributions, or, here, MDDFs, can be used with different trade-offs to obtain the same KB integrals and macroscopic observables.

Despite the differences of MDDFs and RDFs in providing a molecular picture of interactions, the KB integrals computed from MDDFs or RDFs should be equivalent, but only in the limit of very large distances. In Figure 2B we sketch the volumes associated with the “solute domain” computed from the MDDF perspective, in green, or the RDF perspective, in purple. Because ibuprofen is an elongated molecule, the bulk region of the solution is not reached until relatively large isotropic radial distances, but it is reached for shorter minimum-distances, which account for the molecular shape of the solute. This is reflected in the convergence of the KB integrals, shown in the plot of Figure 2B: at about 15 Å from the solute the KB integral computed from the MDDFs starts to oscillate around an apparent molar volume, while that computed from the RDF fails to converge, because at the lengthier dimension of the solute the bulk region of the solution was not yet reached.

The convergence of the KB integrals was evaluated for all simulated systems. For most systems, the integral was observed to stabilize around a plateau from *R* ≈ 25 up to 30 Å, which was qualitatively selected as the convergence threshold. It should be noted that the convergence for systems containing butanediol was significantly poorer, with larger oscillations persisting beyond this distance. Bulk concentrations of the solutions were obtained from the simulations by computing the density of each solvent in the region between 25 and 30 Å from the ibuprofen surface, which is an open system embedded in a larger solvent reservoir, as suggested by Ganguly and co-workers^74,75^ to minimize finite-size effects.

The preferential solvation parameter (Γ) quantifies the competition between the components of the solvent for the interactions with the solute.^11,76–78^ Γ can be computed from the difference between KB integrals of the components of the solvent using

3 $$\Gamma_{2u}\approx \rho_{2}\left[G_{2u}\left(R\right)-G_{3u}\left(R\right)\right]$$

where the subscripts *u*, 2, and 3 refer, respectively, to ibuprofen, the HBD, and the HBA; *2u* and *3u* refer to ibuprofen-HBD and ibuprofen-HBA KBIs.^77–80^ For example, if Γ_2*u*_ is positive, the HBD preferentially solvates ibuprofen. Here, the mean of the last 5 Å of the KB integrals were used to compute the preferential solvation parameter.

### Statistical Validation

The statistical validation of the simulations and equilibration times was performed using the *block_average* function of MolSimToolkit.jl - version 1.32.3. Because statistical correlation times are inherently dependent on the chosen physical descriptor, we explicitly monitored the minimum-distance coordination numbers evaluated at two structurally distinct threshold distances relative to ibuprofen: a short-range cutoff (*r* = 3.5 Å) and a long-range bulk cutoff (*r* = 15 Å). The short-range coordination numbers are used to verify the sampling adequacy of the localized, directional solvation shell. Conversely, the coordination numbers at 15 Å track the particle fluctuations at the bulk boundary used to compute the KB integrals, providing a direct metric for the convergence of the macroscopic thermodynamic properties. The average CNs, integrated correlation times and effective number of uncorrelated samples^81^ of each 30 ns run was obtained, and reported in Table 1.

**Table 1:** Sampling and Correlation Analysis of Coordination Numbers in 30 ns Simulations^a^

system	property	BET (3.5 Å)	BET (15 Å)	HBD (3.5 Å)	HBD (15 Å)
BET - EG	CN	5.8 ± 0.7	84.2 ± 2.8	15.3 ± 0.82	214.3 ± 4.9
	τ_INT_ (avg)	2.2 ± 1.0 ns	3.2 ± 1.4 ns	1.5 ± 0.7 ns	2.6 ± 1.4 ns
	τ_INT_ (range)	(0.9–4.5) ns	(0.8–5.0) ns	(0.4–3.0) ns	(0.5–4.7) ns
	*N*_eff_	17 ± 8	13 ± 10	27 ± 18	17 ± 16
BET - PG	CN	3.8 ± 0.8	70.1 ± 3.6	15.8 ± 0.83	188.4 ± 3.9
	τ_INT_ (avg)	3.2 ± 1.1 ns	3.2 ± 1.4 ns	1.6 ± 0.7 ns	1.7 ± 0.6
	τ_INT_ (range)	(1.6–4.8) ns	(1.8–5.6) ns	(0.8–2.5) ns	(1.2–2.7) ns
	*N*_eff_	11 ± 5	11 ± 4	22 ± 10	19 ± 6
BET - BD	CN	3.7 ± 1.4	59.9 ± 3.5	14.1 ± 0.9	174.9 ± 3.3
	τ_INT_ (avg)	2.20 ± 1.8 ns	2.5 ± 1.7 ns	1.0 ± 0.9 ns	1.7 ± 1.2 ns
	τ_INT_ (range)	(0.6–5.6) ns	(0.8–5.4) ns	(0.1–2.8) ns	(0.6–3.9) ns
	*N*_eff_	25 ± 18	19 ± 14	81 ± 99	27 ± 18

a Each cell displays the average CN, the integrated correlation time τ_INT_, the range of τ_INT_ of the 10 simulation replicas, and the effective number of uncorrelated samples, *N*_eff_.^81^ Short-ranged coordination numbers tend to display faster decorrelation, consistent with the fine convergence of solvent-shell distribution functions. Reported values correspond to the average and standard deviation, and ranges, of the independent replicas for each system.

Table 1 shows that the fluctuations of the CNs computed among replicas are small relative to the simulation averages, and thus, from the perspective of these properties, the independent replicas sampled similar environments, despite the random initial solvent coordinates before equilibration. The average integrated correlation times vary from ∼1 ns to ∼3 ns. These correlation times are used to estimate the number of uncorrelated samples of a time-series, and thus indicate that the equilibration times of 5 ns are generally adequate to decorrelate the CNs from the initial random configurations of the solvent. The average number of effective samples was always greater than 10 per replica, such that more than 100 uncorrelated samples were obtained in the set of 10 replicas for each system.

The integrated correlation times vary between 0.1 ns (for BD at 3.5 Å) and 5.6 ns (for BET with BD at 3.5 Å, and BET with PG at 15 Å). Some very-short correlation times indicate, though, that there are simulations that were trapped in local minima, and sampled only fluctuations of the CN in a basin of the free energy surface. Nevertheless, these simulations display similar average CNs to other samples, and thus do not bias the overall structural analysis of the solution. In general, short-ranged CNs display faster dynamics and a greater number of effective samples than long-ranged CNs, which is consistent with the robust convergence of distribution functions and harder-to-converge KB integrals.

To evaluate the long-range stability of these observables in highly viscous environments, a block-decomposition analysis was performed on the calculated KB integrals. The 30 ns production trajectories for each independent replica were divided into two equal blocks: the first half (0–15 ns) and the second half (15–30 ns). As shown in Table S4 and Figures S7 and S8 of the Supporting Information, the degree of temporal stability varies across systems. For the BET:EG system, the block-averaged KB integral values are statistically equivalent within the standard deviations across replicas, indicating that the long-range solvent structure has reached a stable steady state. For the BET:PG system, the block-to-block variation falls within the standard deviations of both blocks, though absolute KB integral values carry non-negligible uncertainty that should be considered when interpreting quantitative results. For the BET:BD system, larger oscillations persist between blocks, consistent with the slower dynamics of this more viscous and hydrophobic medium, and absolute KB integral values should be treated with corresponding caution. Despite these system-dependent differences in quantitative convergence, the qualitative ranking of preferential solvation and in particular the inversion in the sign of Γ from betaine-preferential in BET:EG to HBD-preferential in BET:BD is reproduced consistently across all replicas and both time blocks, supporting the robustness of the thermodynamic trends derived from the KB theory.

## Results and Discussion

### Structure of Ibuprofen Solvation in DESs

Figure 3 presents the MDDFs for BET and the HBDs in the simulated systems, highlighting distinct distribution patterns at short distances. BET, represented by the orange curves (with different shades depending on the paired HBD species), exhibits two main peaks: one centered at approximately 1.8 Å and another at 2.4 Å. BET contains a carboxylate group capable of forming hydrogen bonds, and the first peak observed in Figures 3A–3C indicates that BET molecules are found at the characteristic hydrogen bond distance.

**Figure 3:**
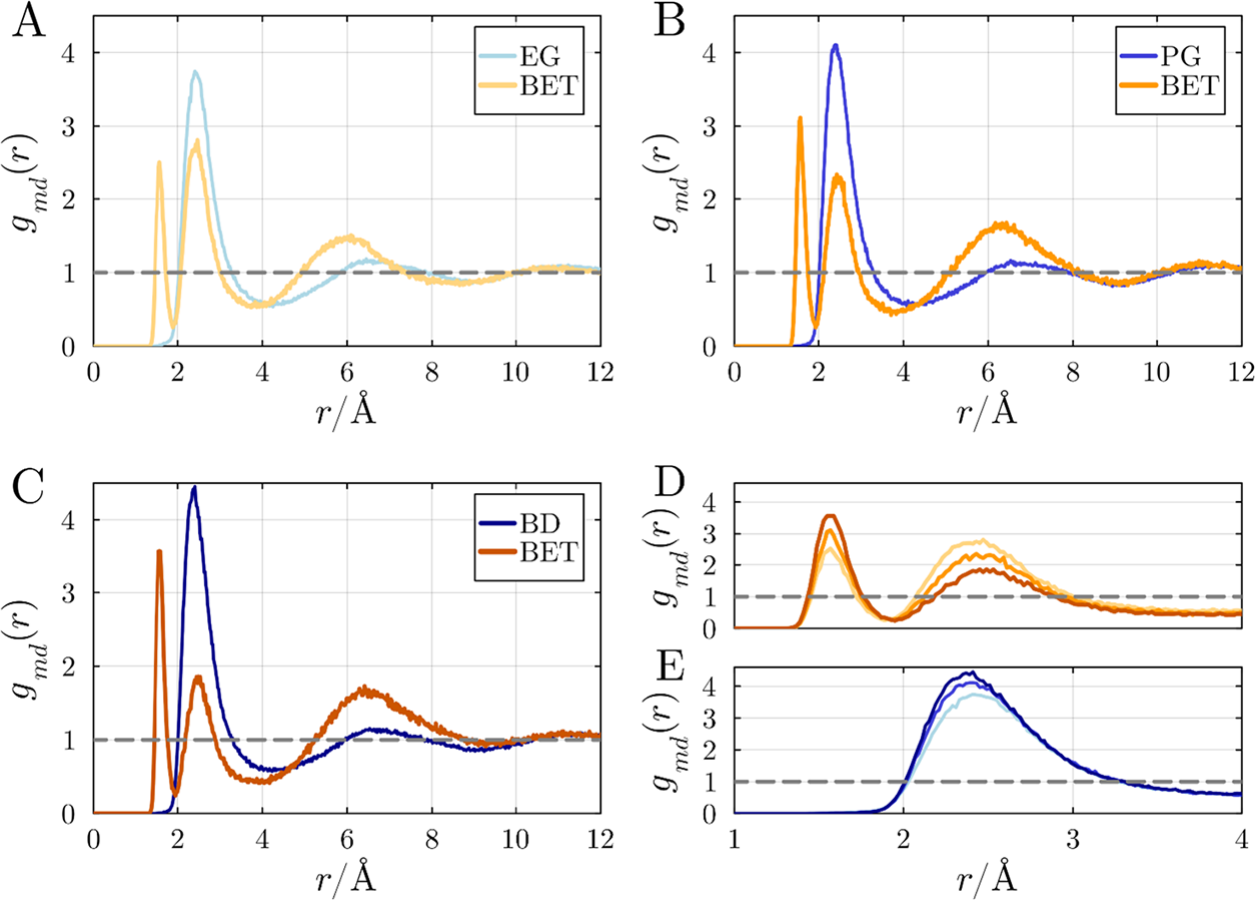
MDDFs between ibuprofen and the DES components in systems containing (A) BET:EG, (B) BET:PG, and (C) BET:BD. Panels (D) and (E) compare the MDDFs of BET and the HBDs, respectively, across the three DES systems, emphasizing differences in local accumulation near the solute. All curves represent the average over 10 independent simulation replicas.

In contrast, the second peak of the BET MDDF at 2.4 Å is not directly associated with specific BET-ibuprofen interactions. Instead, it likely arises from nonspecific (dispersive-like) interactions. Figure 3D compares the BET MDDFs for different alcohol pairings, showing that the relative height of the hydrogen bonding peak is highest when BET is paired with BD, followed by PG, and finally EG. Interestingly, the second solvation peak (∼2.4 Å) exhibits the opposite trend, with the highest relative density observed for BET:EG.

The MDDF for EG, shown in light blue in Figure 3A, exhibits a single prominent peak centered around 2.5 Å. This distance suggests that EG does not form frequent hydrogen bonds with ibuprofen. Therefore, BET is the primary hydrogen bond acceptor to ibuprofen in this system. This pattern is consistent across all systems studied. Figure 3E compares the first solvation peaks for different HBD species, with BD showing the highest accumulation around ibuprofen, followed by PG and EG. BET dominates the hydrogen bonding interactions with ibuprofen. However, with the increasing alkyl chain length of the alcohol HBDs, the nonspecific interactions (e.g., hydrophobic) of the HBDs with ibuprofen become more pronounced than those of BET with ibuprofen.

Figure 4 presents the decomposition of the MDDFs for each chemical group of the DESs, using the BET:EG system as a representative example. Figure 4A displays the MDDF decomposed by the atomic groups for EG. The dominant contributions to the first peak arise from the CH_2_ groups of the EG molecule, indicating a preferential proximity of the hydrophobic ethylene backbone to the solute. Notably, the hydroxyl (OH) group contributes less significantly to the peak, suggesting a reduced role in establishing direct interactions with ibuprofen compared to the aliphatic segments. Figure 4B shows the MDDF decomposition for betaine, resolved into contributions from the carboxylate group, the methylene group (CH_2_), and the trimethylammonium group [N­(CH_3_)_3_]^+^. The first solvation peak arises exclusively from interactions involving the carboxylate group, confirming its role as the hydrogen bond acceptor. The second peak originates from nonspecific contacts between the [N­(CH_3_)_3_]^+^ group and the ibuprofen surface, consistently with the more distant positioning observed in the total MDDF.

**Figure 4:**
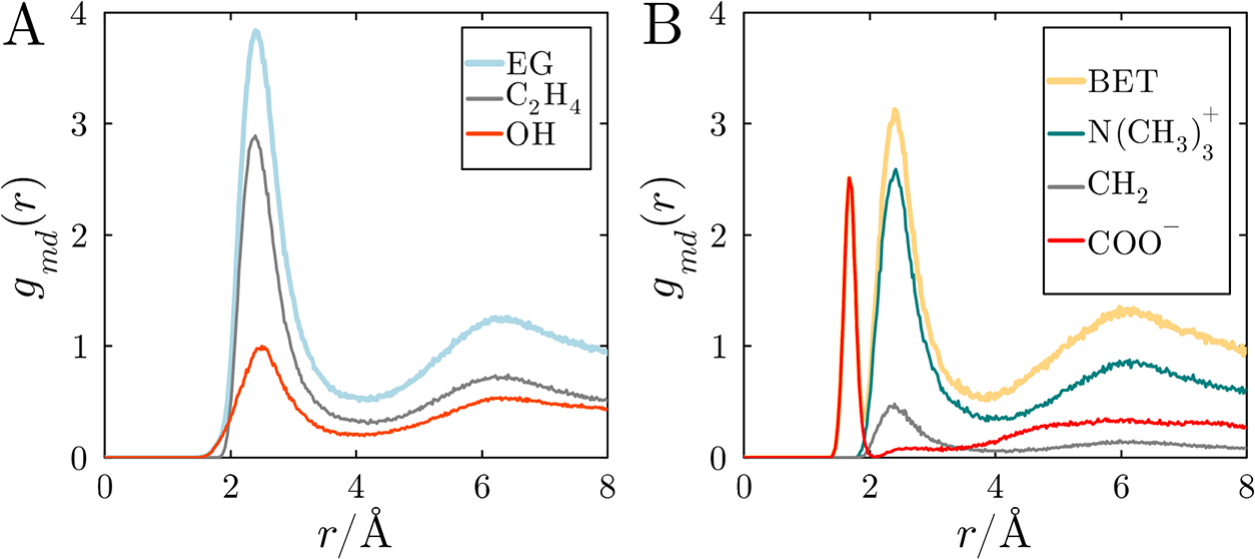
Decomposition of MDDFs of the solvent components in the BET:EG system. A) Decomposition of the EG MDDF by chemical functional groups. The CH_2_ groups contribute most significantly to the first solvation peak, indicating preferential accumulation near the ibuprofen surface, while the OH group plays a comparatively minor role. B) Decomposition of the betaine MDDF into its functional groups: COO^–^, CH_2_, and [N­(CH_3_)_3_]^+^. The first peak is entirely attributed to the COO^–^ group, confirming its role as a hydrogen bond acceptor, whereas the second peak is primarily associated with the [N­(CH_3_)_3_]^+^ moiety.

To further elucidate the molecular features governing solvation, MDDFs were also decomposed by functional groups of the ibuprofen molecule, namely CH_2_CH­(CH_3_)_2_ (isobutyl side chain), C_6_H_4_ (aromatic ring), CHCH_3_ (olefinic methyl), and COOH (carboxylic acid). Among these, only the COOH group is expected to participate in strong directional interactions such as hydrogen bonding, while the remaining groups primarily contribute through nonspecific dispersive interactions.

Figure 5 presents the decomposition of the MDDFs into the contributions from different functional groups of ibuprofen. The decomposition essentially divides the ibuprofen molecule into 3 parts: the carboxylic acid (COOH), the aromatic ring group (C_6_H_4_), and the isobutyl with the olefinic CHCH_3_ (C_6_H_13_). As discussed before, in Figure 3, the MDDFs show that the alcohols do not interact with the solute via hydrogen bonds. The decomposition in Figure 5A displays that, using the illustrative example of EG, the ibuprofen-EG interactions are through the nonpolar C_6_H_13_ group, represented by the gray curve. There are also contributions from C_6_H_4_ and COOH, but at distances corresponding to dispersive interactions.

**Figure 5:**
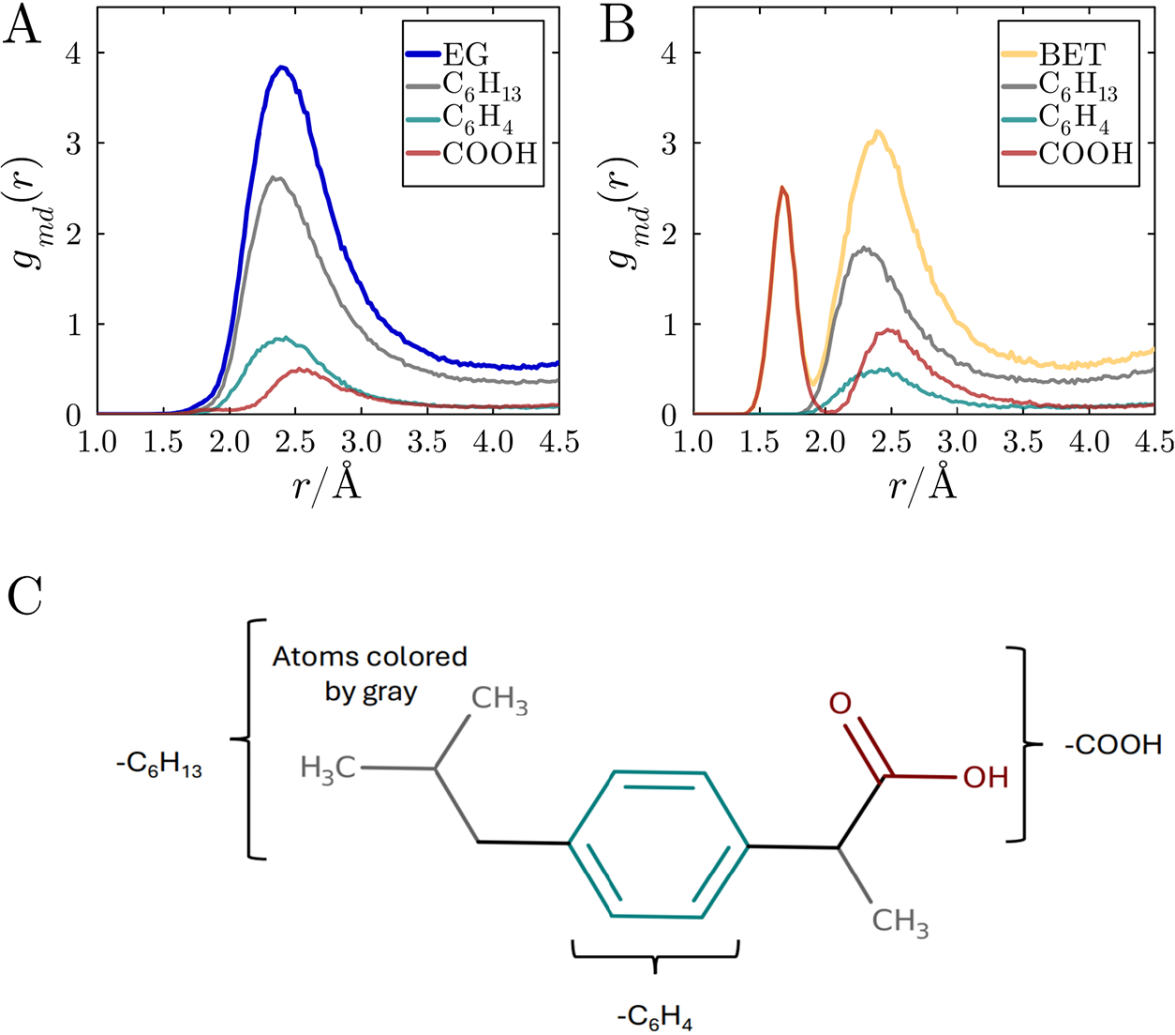
Decomposition of MDDFs in terms of ibuprofen chemical groups. (A) Decomposition of the EG MDDF, hydrophobic sites from ibuprofen are those that interact more with EG. (B) The first peak of the betaine MDDF shows contributions from betaine atoms around the COOH group of ibuprofen. The second peak has contributions from the COOH group and significant contributions from the isopropyl group (gray). (C) representation of ibuprofen molecule with colors correspondent to the MDDFs decomposition.

The first peak in the betaine MDDF (Figure 5B) arises exclusively from interactions with the COOH group of ibuprofen, consistent with hydrogen bonding. In contrast, the second peak presents a more heterogeneous composition. While the COOH group still contributes to this region, the most prominent contribution originates from the aliphatic C_6_H_13_ group (gray curve), such that these nonspecific interactions occur primarily between betaine and the hydrophobic segments of the solute. A smaller, yet discernible, contribution from the aromatic ring is also observed.

The significant participation of hydrophobic groups in these interactions can be rationalized by the molecular composition of ibuprofen, which is predominantly nonpolar. This structural characteristic increases the probability of surrounding solvent molecules, particularly EG and BET, being distributed near the hydrophobic moieties of the solute. For example, the preferential localization of EG molecules around the isopropyl and aromatic groups reflects mainly dispersive, van der Waals-type interactions. Additionally, the presence of BET at approximately 2.2 Å from these hydrophobic groups reinforces the role of nonpolar interactions in defining the organization of the solvation shell.

The structural analysis from the MDDFs can be qualitatively compared with experimental macroscopic characterizations of active pharmaceutical ingredients (API)-based eutectic systems to improve the understanding of the solvation mechanism in the DESs simulated here. For instance, experimental results from Nica et al. and Kefayati and Haghtalab suggest that the solvation of acidic APIs is primarily governed by the hydrogen-bond basicity (β) of the solvent components.^82,83^ Here, the ibuprofen carboxylic group establishes specific interactions with the zwitterionic HBA (Figure 5B), acting as a hydrogen-bond donor to the basic betaine carboxylate site. Furthermore, Farooq et al. observed that diol-based systems exhibit high dipolarity but relatively weak dispersive interactions, which is qualitatively consistent with our finding that smaller diols like ethylene glycol are largely excluded from the first solvation shell.^84^ The shift we observe toward hydrophobic control as the alkyl chain length increases represents the mechanistic transition where these dispersive forces begin to dominate, mirroring experimental trends where hydrophobic natural deep eutectic solvents (HNADES) achieve significantly higher solubility enhancements by effectively shielding the drug’s hydrophobic phenyl ring.^82,85^

The EG distributions display broader peaks, with the most significant contributions arising from its methylene (CH_2_) groups (Figure 4A). This suggests that EG preferentially interacts through van der Waals contacts, and possibly through hydrogen bonding with other solvent molecules, rather than with ibuprofen directly. Such interactions could also be cooperative, mediated by the extended hydrogen-bond network characteristic of DESs.

To investigate possible autocorrelations of betaine at the surface of ibuprofen, we computed the distributions of the carbonyl oxygen and nitrogen independently, relative to ibuprofen, as shown in Figure 6. The intention was to investigate successive layers of opposing charges promoting long-range organization of betaine. If this were the case, the peaks and dips of these distributions should be highly correlated.

**Figure 6:**
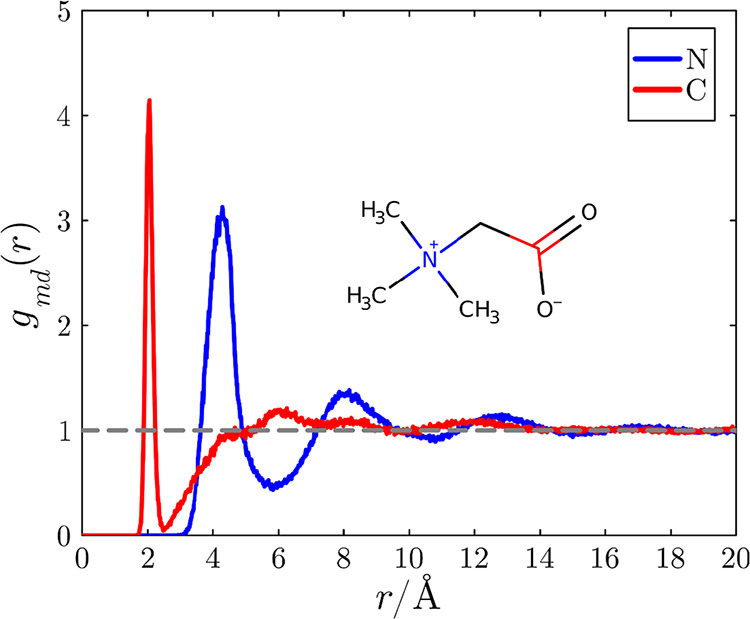
Atomic minimum-distance distribution functions (MDDFs) for carbon atoms from the COO^–^ group (red) and for nitrogen atoms (blue) in betaine, in the BET–EG system.

The carboxyl-carbon initial peak, at ∼2 Å, is followed by the nitrogen peak at ∼4.2 Å, but in Figure 4B (red and green curves) we see that these are direct hydrogen-bonds and nonspecific interactions mediated by other betaine atoms of the same molecule. A small peak of the carbon MDDF at 6 Å appears to follow the large N peak and is possibly associated with charge correlations with the first N peak. Finally, the second N peak at 8.5 Å appears to follow the C peak at 6 Å, consistently with charge correlations, but the shapes and height of the peaks do not match, indicating that these correlations are weak. The second solvation shell observed in the N atom distribution likely originated from other correlations in the system, for example, with nonspecific interactions with intermediating EG molecules.

### Thermodynamics of Solvation Using Kirkwood-buff Integrals

Figure 7 presents the KB integrals for all DESs combinations simulated. As discussed previously, DESs are characterized by their high viscosities, which can reach values several orders of magnitude greater than that of water. This elevated viscosity is associated with markedly slower molecular dynamics, meaning that the translational and rotational motions of molecules are significantly hindered.

**Figure 7:**
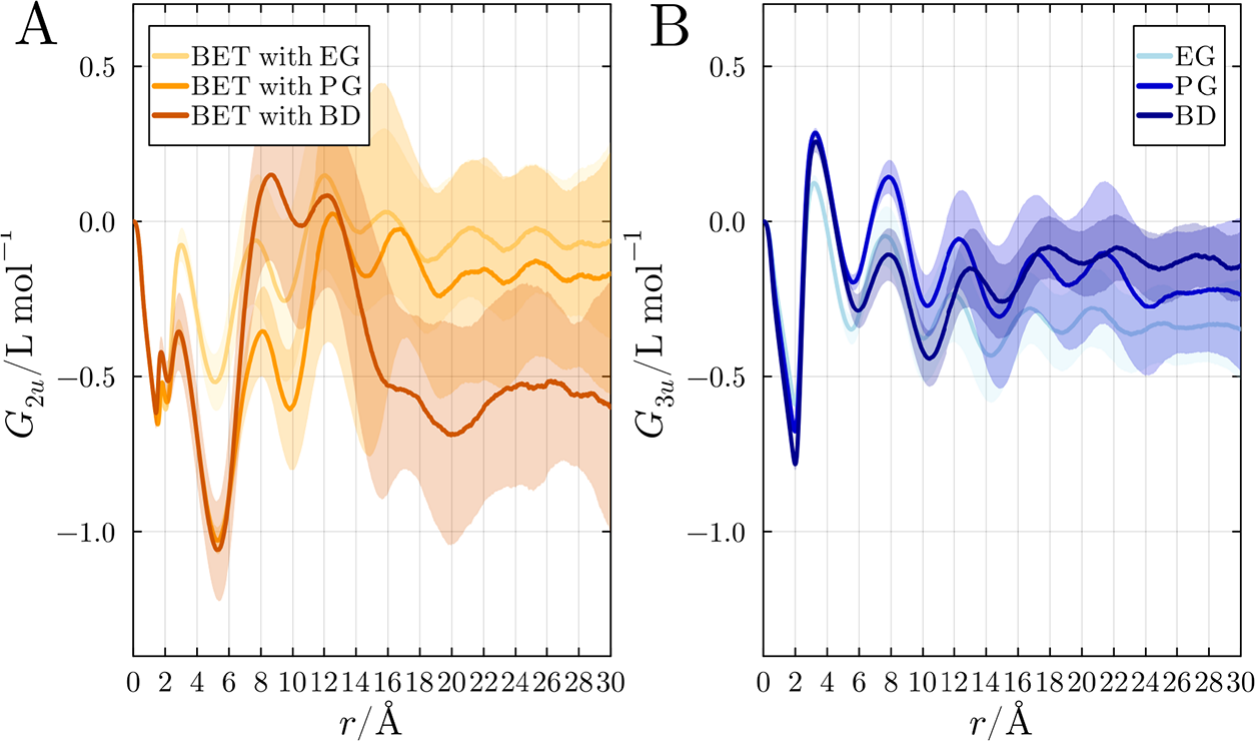
KB integrals for the HBDs (*G*_*2u*_) and HBAs (*G*_*3u*_) of all DESs simulated. The initial decrease in the curves reflects the steric exclusion of ibuprofen from the immediate vicinity. (A) The subsequent rise is associated with short-range hydrogen-bond interactions between betaine and the solute. (B) The curves in different shades of blue illustrate the behavior of the HBDs, which exhibit minimal short-range accumulation, indicating the absence of hydrogen bonding with ibuprofen. The shadows around the solid curves represent the standard error calculated with the 10 simulated replicas of each system.

Such sluggish dynamics imply that sampling efficiency in DESs is much harder than for typical molecular liquids. As detailed in the Materials and Methods section, the calculation of KB integrals is inherently challenging due to their dependence on the surface element *S­(r)*, which requires adequate convergence of spatial correlations over long distances. In the present simulations, the elevated viscosity of the DESs, combined with their intrinsic hydrogen-bonding networks and electrostatic interactions, promotes the occurrence of long-range structural correlations.

From 0 to 10 Å, the KB integral curves reflect local solvation patterns that correspond to the features observed in MDDFs. The light-orange curve in Figure 7, representing betaine in the BET:EG system, initially exhibits a decrease at short distances. This negative deviation is attributed to the steric exclusion of betaine molecules from the immediate vicinity of ibuprofen. Following this initial exclusion zone, a small positive deviation is observed between approximately 1.8 and 2.1 Å, corresponding to the short-range hydrogen-bonding interactions between the carboxylic acid group of ibuprofen and the carboxylate group of betaine.

While the MDDFs indicate that hydrogen bonding is a key short-range interaction, particularly between betaine and ibuprofen,they also reveal that ethylene glycol does not engage significantly in hydrogen bonding with the solute. However, a closer examination of the KB integral profile suggests that these short-range hydrogen bonds contribute only modestly to the total accumulation of betaine near ibuprofen. The cumulative KB integral for betaine continues to increase at longer distances, indicating that the dominant contribution to the solvation structure arises from long-range interactions. This observation suggests that, although hydrogen bonds initiate the local structuring of betaine around ibuprofen, the accumulation reflected in the KB integral is primarily governed by long-range correlations arising from extended electrostatic interactions and the cooperative organization of the solvent network in the high-viscosity DES environment.

To evaluate the temporal stability of these long-range observables, a block-decomposition analysis was performed by dividing each 30 ns production trajectory into two consecutive 15 ns intervals across all 10 independent replicas (Table S4, Figures S7–S8). For the BET:EG system, the KB integral values from both time blocks are statistically equivalent within the standard deviations across replicas. For the BET:PG system, the block-to-block variation in the HBA KB integral falls within the standard deviations of both blocks, though the absolute values carry non-negligible uncertainty that should be considered when interpreting quantitative results for this system. For the BET:BD system, larger oscillations persist at long-range, consistent with the slower dynamics of the more viscous and hydrophobic medium, and absolute KB integral values should be interpreted with corresponding caution. The block decomposition shows that the qualitative trends and in particular the preferential solvation inversion are stable across time blocks, while absolute KBI values, especially for BET:PG and BET:BD, carry non-negligible uncertainty that we have explicitly acknowledged. Therefore, while definitive quantitative conclusions from the absolute KB integral values should be treated with caution, the relative rankings and the direction of preferential solvation across the HBD series are robust and reproducible across all replicas and both time intervals sampled.

The KB integrals for BET suggest the following qualitative order of preferential association with ibuprofen: BET:EG > BET:PG > BET:BD. Although all HBA KBI values are negative, reflecting a net long-range depletion of betaine relative to bulk in all systems, betaine is least depleted when paired with EG, indicating that its affinity for ibuprofen is greatest in this system. This is consistent with the strong and specific COOH···COO^–^ hydrogen-bonding interaction identified in the MDDFs, which creates a local enrichment at short-range that is not fully captured by the sign of the long-range KBI alone.

Conversely, the KB integrals for the HBD components show a reversed trend, with BD exhibiting the largest long-range accumulation, despite being associated with the lowest BET accumulation. While the differences in the KB integrals or HBDs and HBAs appear small and within the simulated uncertainties, they are actually reflective of significant variations in preferential solvation across the systems, because the bulk molar density of the DESs is greater for DESs with smaller aliphatic chains, as shown in Table 2. This will be discussed in the next section.

**Table 2:** Bulk Concentration of the Hydrogen Bond Acceptors (*C*_*HBA*_) and Donors (*C*_*HBD*_) in the Simulated DESs^a^

system	*C*_*HBA*_ (mol L^–1^)	*C*_*HBD*_ (mol L^–1^)
BET:EG	3.79 ± 0.04	11.07 ± 0.07
BET:PG	3.21 ± 0.07	9.33 ± 0.09
BET:BD	2.52 ± 0.09	7.55 ± 0.08

a Errors represent the standard error calculated from 10 different replicas for each system.

### Preferential Solvation

The preferential solvation parameter, Γ, quantifies the relative affinity of solvent components to the solute (eq 3). It is defined based on the change in the chemical potential of the solute upon the addition of cosolvent. Figure 8 shows the preferential solvation parameter Γ for systems composed of BET (Γ_*3u*_) combined with the different HBDs (Γ_*2u*_): EG, PG, and BD. A positive Γ_*3u*_ for BET (1.04), in contrast with that for EG (−3.28), indicates that ibuprofen is preferentially solvated by betaine, such that interactions between BET and ibuprofen are more favorable than those between EG and ibuprofen. The greatest preferential solvation by betaine occurs in the BET:EG system. This behavior can be attributed to BET’s ability to establish specific interactions with the carboxylic group of ibuprofen, such as hydrogen bonds.

**Figure 8:**
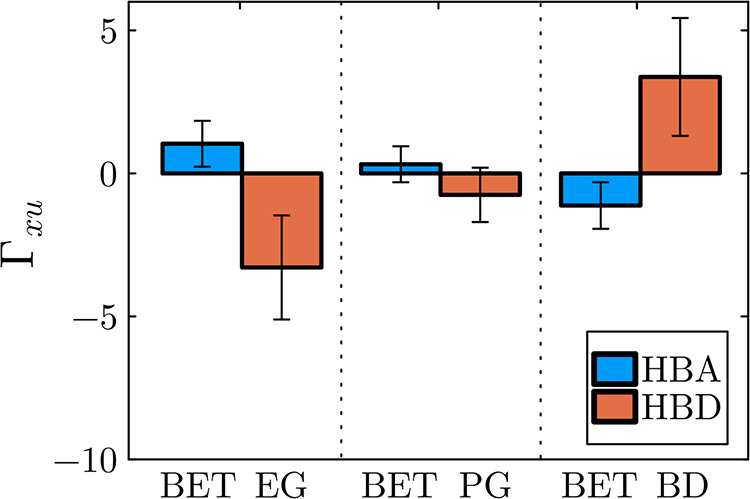
Preferential solvation parameters (Γ_*xu*_, where *x* are the BET, in blue, or the HBDs, in orange) of solvent components for the systems BET:EG, BET:PG, and BET:BD. Positive preferential solvation indicates preferential accumulation of the component around ibuprofen, whereas negative values reflect preferential depletion. The BET–EG system shows the strongest preferential solvation by BET. As the HBD’s alkyl chain length increases, solvation shifts and preferential solvation by the HBD become dominant.

As the alkyl chain length of the HBD increases from EG to PG and BD, there is a shift in solvation preference. The preferential solvation parameter of ibuprofen by BET decreases progressively, while that of the HBD becomes less negative for PG and turns positive for BD. Therefore, the preferential solvation of ibuprofen by the HBD increases with the hydrophobicity of the alcohol. Additional factors, such as the local solution structure and cooperative interactions between the BET and the HBD, may play a significant role in the solvation behavior of ibuprofen.

## Conclusion

This study explored the solvation of ibuprofen in three deep eutectic solvents (DESs) composed of betaine and different hydrogen-bond donors (ethylene glycol, propylene glycol, and 1,2-butanediol), using polarizable molecular dynamics simulations. By combining minimum-distance distribution functions, Kirkwood-Buff integrals, and preferential solvation parameters, we analyzed both the local and long-range solute–solvent interactions in these highly viscous and structurally complex systems.

The simulations showed that BET interacts via a strong hydrogen bonding with the carboxylic group of ibuprofen, while alcohols, particularly EG, are preferentially excluded from ibuprofen. MDDF decompositions revealed that hydrophobic regions of ibuprofen promote the accumulation of both betaine and the HBDs, depending on their chain length and polarity. Notably, preferential solvation shifted from betaine to HBD as the alkyl chain of the donor increased, reflecting enhanced hydrophobic interactions in the BET–BD system.

Kirkwood–Buff theory proved valuable in capturing solute–solvent affinities beyond the first solvation shell, offering a thermodynamic perspective that complements the spatial resolution of MDDFs. However, its application in DESs also revealed intrinsic challenges: The high viscosity of DESs slows molecular diffusion, leading to long convergence times for the solvent structure and, thus, of KB integrals. Nevertheless, qualitative trends in KB integral behavior were still informative, particularly for the BET:EG and BET:PG systems where block-decomposition analysis confirmed temporal stability of the long-range structure. For the BET:BD system, while absolute KBI values carry greater uncertainty due to the slower dynamics of this more hydrophobic medium, the direction of preferential solvation remains consistent across replicas and time blocks. The KB integrals regime observed after ∼20 Å allowed for comparison across systems and revealed preferential accumulation patterns consistent with the hydrophobic character of the solvent.

These insights underscore that, while KB theory remains a powerful tool for understanding solvation in nonideal systems, its quantitative application in DESs demands careful consideration of simulation length, temperature, and statistical convergence. The approaches demonstrated here can support future investigations into drug solubility, solute partitioning, and the molecular design of DESs for pharmaceutical and biomolecular applications.

## Supplementary Material



## Data Availability

Scripts, force field parameters, and molecular structures to replicate the simulation boxes and perform the molecular dynamics simulations used are available at 10.5281/zenodo.19269578.

## Notes

Box dimensions, number of species, and experimental and post-NPT densities for all simulated systems (Table S1). Nonbonded parameters used for betaine and ibuprofen in the CL&Pol force field (Tables S2 and S3, respectively). Minimum-distance distribution function (MDDF) decompositions for BET:PG and BET:BD systems, detailing atomic contributions from both HBDs and betaine (Figures S1–S2). Zwitterionic structure of betaine and methodology for partial charge derivation (Figure S3), and chemical structure of ibuprofen (Figure S4). Temperature and potential energy during equilibration (Figure S5). System density during equilibration (Figure S6) and block decomposition of KB integrals (Figures S7 and S8). Estimated converged values for KB integrals in the first and last 15 ns of the production step of each replicate of the systems simulated (Table S4). Details on force field construction, implementation of Drude dipoles, and damping functions used to prevent polarization instabilities are also provided in the section *“Additional details of the simulations”* (PDF)
