# Supplementary material for: From Hydrogen Bonding to Hydrophobic Control: The Shifting Solvation Mechanism of Ibuprofen in Deep Eutectic Solvents
Source: J Phys Chem B. 2026 Jun 20;130(29):7388–400. doi: 10.1021/acs.jpcb.5c08530 (PMC13403312; doi:10.1021/acs.jpcb.5c08530)
Supplement: Supplementary file 1 [file jp5c08530_si_001.pdf]

Supporting Information

From Hydrogen Bonding to Hydrophobic Control: The Shifting Solvation Mechanism of Ibuprofen in Deep Eutectic Solvents

Vinicius Piccoli[1], *Agílio Pádua*[2,*], *Leandro Martínez*[1,*]

1 - Institute of Chemistry and Center for Computing in Engineering & Science, Universidade Estadual de Campinas (UNICAMP), 13083-861 Campinas, SP, Brazil

2 - Chemistry Laboratory, École Normale Supérieure (ENS) de Lyon, 46, allée d'Italie 69364 Lyon Cedex 07, France.

Contents:

1) **Table S1:** Simulation box dimensions, experimental and post-NPT densities, and number of species used in each system.
2) **Table S2:** Non-bonded force field parameters used for betaine in the CL&Pol model.
3) **Table S3:** Non-bonded force field parameters used for ibuprofen in the CL&Pol model.
4) **Figures S1–S2:** Minimum-distance distribution function (MDDF) decompositions for BET:PG and BET:BD systems, including atomic group contributions from HBDs and betaine.
5) **Figure S3:** Zwitterionic structure of betaine used in the simulations.
6) **Figure S4:** Chemical structure of ibuprofen used in the simulations.
7) **Technical Notes:** Parameterizing details for betaine, implementation of Drude polarizability in the CL&Pol force field, and discussion of damping functions used to ensure simulation stability.
8) **Control parameters convergence during equilibration**: Time-dependent profiles displaying the replica-by-replica relaxation and stabilization of the system density (ϱ), potential energy (E_{pot}), and temperature (T) across the ten independent simulation runs for each mixture, showcasing thermodynamic stability before production sampling.
9) **KB integrals block decomposition:** Figures S7 and S8 displaying the running Kirkwood–Buff integrals calculated independently for the hydrogen bond donor (HBD) and acceptor (HBA) species during the first half (0–15 ns) and the last half (15–30 ns) of the production phase across all independent

replicates. Table S4 reports the ensemble mean values and standard deviations of the macroscopic KB integrals, calculated by averaging the stabilization plateaus between 25.0 and 30.0Å from the solute surface.

Table S1. Box dimensions, experimental and post-NPT production densities, and components used to construct the simulation boxes. Densities and proportions were referenced from the experimental study by Rodrigues et al. [1].

Systems (1:3 proportion in terms of number of species)	Box sides / Å	Experimental density (g cm^{-3})	Calculated density after NPT equilibration (g cm^{-3})	Number of HBAs species	Number of HBDs species
BET:EG	81.12	1.13	1.129 ± 0.001	1200	3600
BET:PG	79.44	1.07	1.077 ± 0.002	945	2835
BET:BD	80.70	_	1.033 ± 0.001	823	2469

Solvation structure

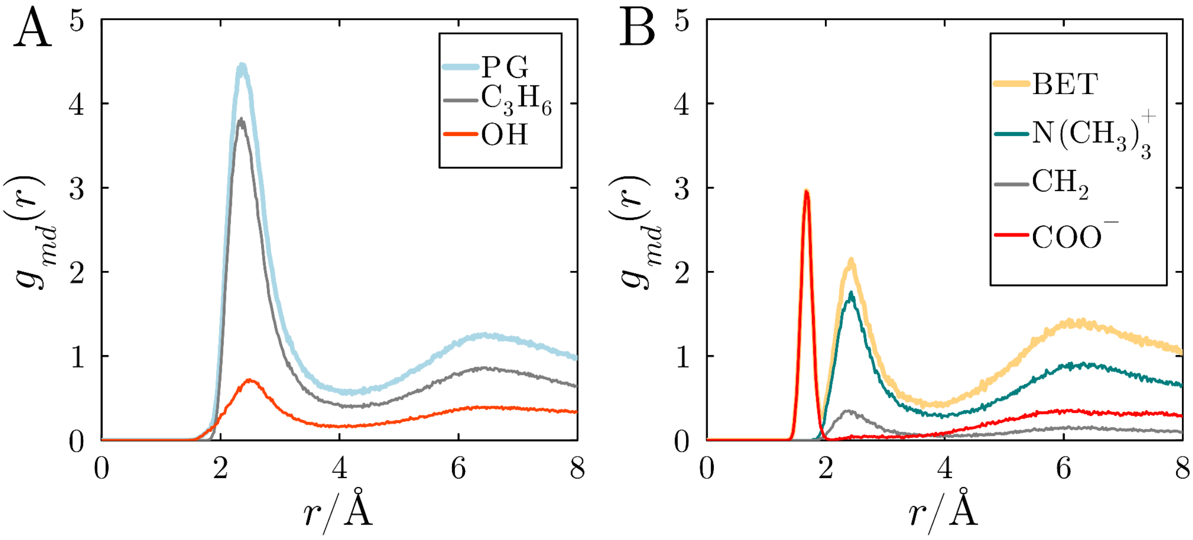

Figure S1. Decomposition of the minimum-distance distribution functions (MDDFs) for the BET:PG system. A) Contribution of propylene glycol (PG) atoms to the MDDF, highlighting the dominant role of CH and CH_2 groups near the ibuprofen surface, with a secondary contribution from the OH groups. B) Decomposition of the betaine MDDF by atomic group (COO^-, CH_2, $[N(CH_3)_3]^+$), showing a similar interaction pattern to that observed in the BET:EG system. Results are averaged over 10 simulation replicates.

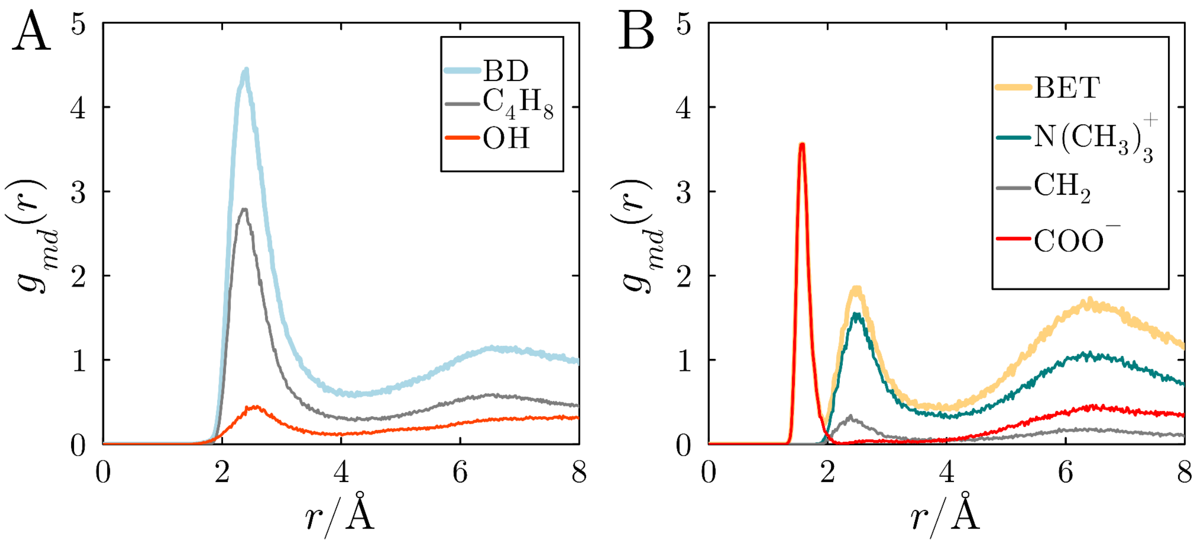

Figure S2. Decomposition of the BET: BD system's minimum-distance distribution functions (MDDFs). A) Contribution of 1,2-butanediol (BD) atoms to the MDDF, revealing a dominant accumulation of CH_2 atoms near ibuprofen, with comparatively lower contribution from OH groups. B) Decomposition of the betaine MDDF into COO^-, CH_2, and $[N(CH_3)_3]^+$ groups, showing consistent trends with other DES systems. All data represent averages over 10 independent simulations.

Additional details of the simulations

All molecular dynamics (MD) simulations were performed using the **CL&Pol polarizable force field**. This force field is based on OPLS-AA parameters and is designed to be extendable to new compounds via a fragmentation approach, ensuring parameter transferability.[2–5] Most parameters for the simulated systems were obtained from the publicly available CL&Pol GitHub repository `clandpol`. This included parameters for the deep eutectic solvent (DES) hydrogen bond donors (HBDs): **ethylene glycol (EG)**, **propylene glycol (PG)**, and **1,2-butanediol (BD)**, which were adapted from existing CL&Pol and OPLS-AA fixed-charge models. However, new parameters were required for components not present in the repository: the hydrogen bond acceptor (HBA) betaine (BET) and the solute ibuprofen (IBU). The force field input files for all components were generated using the fftool program.[8]

Parameters for the nitrogen-containing fragment of betaine were not available in the CL&Pol library. The zwitterionic structure of betaine (Figure S3) was constructed using Avogadro software.[6]

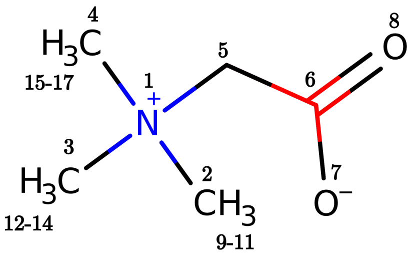

Figure S3. Zwitterionic structure of betaine used in the simulations.

Atomic partial charges were subsequently derived from a geometry optimization performed with Gaussian using the **B3LYP/6-31G(d)** level of theory.[7] The final non-bonded (charge and Lennard-Jones) parameters for betaine are listed in Table S2.

Table S2. Charges and Lennard-Jones Parameters for Betaine.

	Label	AtomType (OPLS)	Mass (u)	Charge (e)	Sigma (Å)	Epsilon (kJ mol^{-1})
1	N4B	N3	14.007	0.340	3.25	0.71100
2-4	C1	CT	12.011	-0.17	3.50	0.27614
7-8	O2	O2	15.999	-0.80	2.96	0.87864
6	CO2	CO	12.011	0.70	3.75	0.43932
5	C2C	CT	12.011	-0.22	3.50	0.27614
18-19	HC	HC	1.008	0.06	2.50	0.12552
9-17	H1	HC	1.008	0.13	2.50	0.12552

Parameters for ibuprofen were also developed using the CL&Pol fragmentation methodology. This approach involves decomposing a large molecule into smaller, chemically consistent fragments for which parameters can be derived. This method maintains the transferability of functional-group parameters across different molecules. Ibuprofen was divided into three fragments: **acetic acid**, **2-methylpropane**, and **toluene**, as depicted in Figure S4.

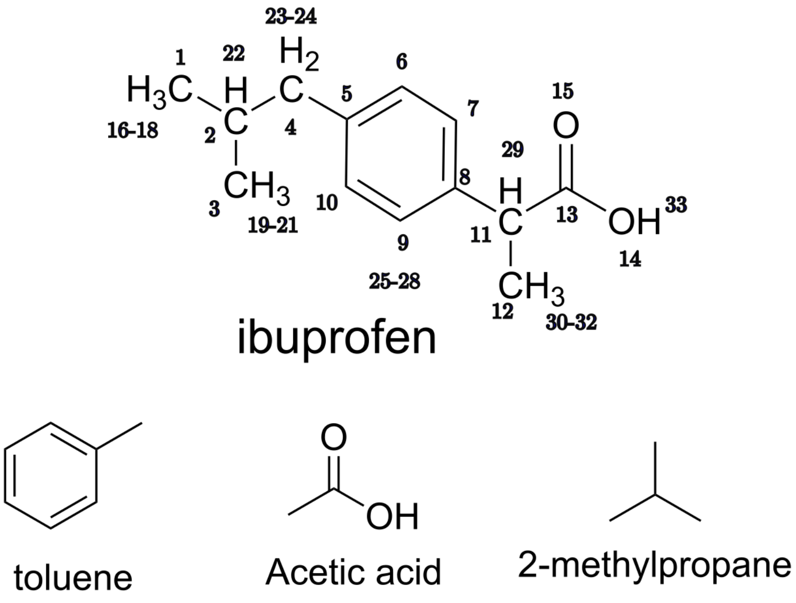

Figure S4. Fragmentation of ibuprofen into acetic acid, 2-methylpropane, and toluene for parameter derivation.

The new fragments were parametrized to obtain atomic charges using the same protocol as for betaine (B3LYP/6-31G(d) geometry optimization). This procedure has been discussed in detail in previous publications describing the development of CL&Pol. The final parametrized charges and Lennard-Jones parameters for ibuprofen are provided in Table S3.

To introduce polarizability into the system, we incorporated Drude dipoles following the procedure outlined in CL&Pol,[9] by adapting the fixed-charge model. Each polarizable atom, mainly heavy atoms such as carbon, oxygen, and nitrogen, is associated with a Drude particle of 0.4 Da, tethered through a harmonic spring with a force constant kD = 4184 kJ mol^{-1}. The magnitude of the opposite charges on the particles constituting the Drude dipole is determined by the polarizability of the corresponding atom, calculated using the formula $\alpha = q_D/k_D$.[9,10] Hydrogen atoms, due to their negligible mass and electron density, are treated as nonpolarizable. However, their polarizability is accounted for by adding the respective value to the heavy atoms bonded to them.

Table S3. Charges and Lennard-Jones Parameters for Ibuprofen

	Label	AtomType (OPLS)	Mass (u)	Charge (e)	Sigma (Å)	Epsilon (kJ mol^{-1})
1, 2, 25	CT	CT	12.011	-0.18	3.50	0.27614
3, 24	CT3	CT	12.011	-0.06	3.50	0.27614
4-9, 11, 13, 14, 26-28, 30	HC	HC	1.008	0.06	2.50	0.12552
10	CT2	CT	12.011	-0.12	3.50	0.27614
12	CTA	CA	12.011	-0.115	3.55	0.29288
15-19	CA	CA	12.011	-0.115	3.55	0.29288
20-23	HA	HA	1.008	0.115	2.42	0.12552
29	COH	C	12.011	0.331	3.75	0.43900
31	OHC	OHC	15.999	-0.25	3.00	0.71100
32	OC3	O_3	15.999	-0.269	2.96	0.87900
33	HO	HO	1.008	0.418	0	0

Short-range interactions between Drude particles are attenuated using Thole functions, with a universal parameter a = 2.6. Additionally, Tang-Toennies' damping functions are applied to mitigate electrostatic interactions between the hydroxyl hydrogen atoms of the hydrogen-bond donors and Drude dipoles in short ranges.[11,12] These damping functions prevent instabilities in the trajectories of the Drude dipoles, commonly referred to as polarization catastrophes, which occur when the point-charge approximation becomes inadequate at short distances.[13]

To accurately model the short-range interactions between charges and induced dipoles and avoid the polarization catastrophe, a custom non-bonded force, referred to as CoulTT, was implemented. This force introduces a charge-dipole

damping function, defined by Equation (S1):

$$E_{damp} = \frac{q_1 q_2}{4\pi\varepsilon_0 r}\left(- ce^{-br}\left(1 + br + \frac{(br)^2}{2} + \frac{(br)^3}{6} + \frac{(br)^4}{24}\right)\right), \quad \text{(S1)}$$

where q_1 and q_2 are the charges, r is the distance between particles, c is a scaling factor, and b is a dumping parameter. We applied the dumping function solely to the charge-dipole interactions between hydrogens and very electronegative atoms, which in the simulated systems extended to oxygens, with parameters $c = 1$ and $b = 52$. This damping function was integrated into the OpenMM simulation using the `CustomNonbondedForce` class, enabling the inclusion of the defined functional form in the total potential energy of the system. An example of the OpenMM implementation used in this work, demonstrating the setup of this `CustomNonbondedForce` and the application of the CoulTT damping, is available at https://github.com/paduagroup/pol_openmm.

Control variables during equilibration

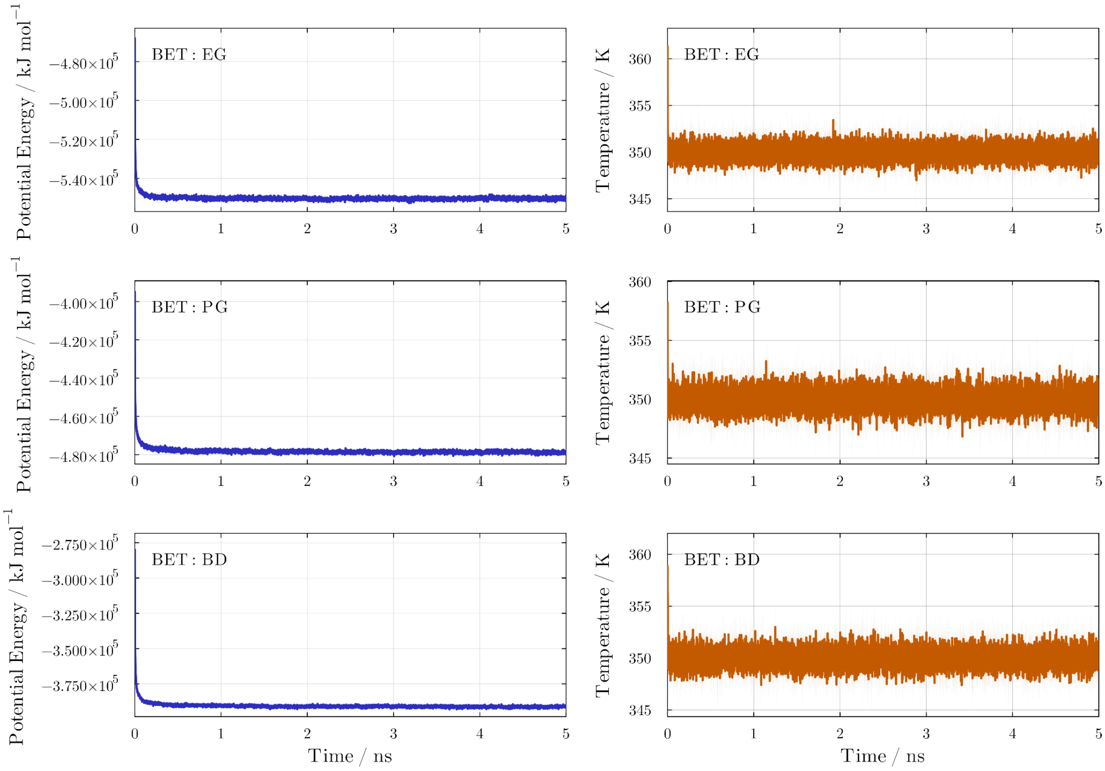

Figure S5. Time evolution of the system density (ϱ) and potential energy (Epot) during the equilibration and production phases for the simulated deep eutectic solvent mixtures. The blue curves represent the trajectory of the potential energy during the initial energy minimization stage, and all subsequent curves display the ensemble mean values calculated across the ten independent simulation replicas for each system. The orange profiles track the temperature dependence and thermal stability maintained throughout the complete simulation timeline.

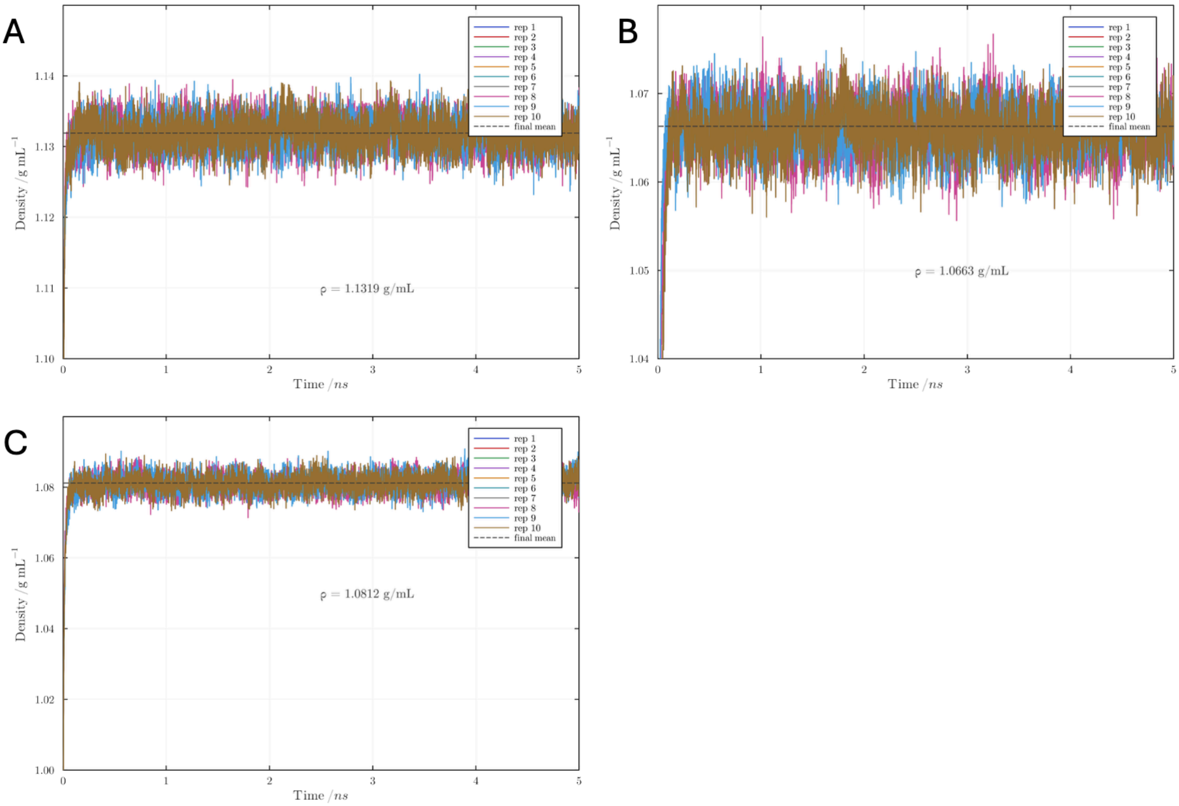

Figure S6. Time-dependent system density profiles during the equilibration phase for the systems simulated at 353 K, detailing the replica-by-replica trajectory across the ten independent simulation runs. The individual curves showcase the exceptional structural stability, rapid volume relaxation, and tight density stabilization achieved for each independent replica. The resulting ensemble mean densities calculated for each deep eutectic solvent system are: (A) 1.1319 g/mL for BET-EG, (B) 1.0812 g/mL for BET-PG, and (C) 1.0663 g/mL for BET-BD.

Convergence of the KB integrals

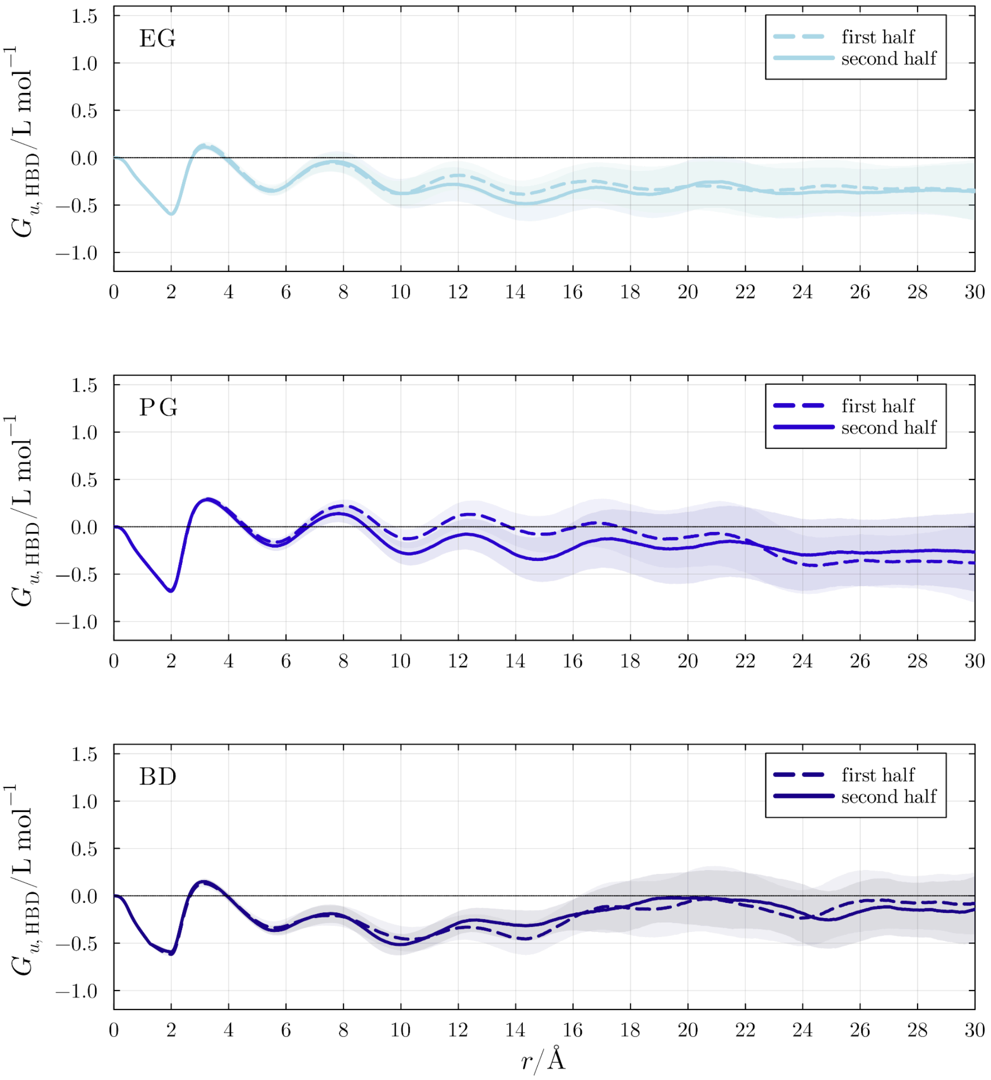

Figure S7. Long-range spatial convergence and block-decomposition analysis of the Kirkwood–Buff integrals for the hydrogen bond donors (HBD) species across the simulated deep eutectic solvents. To evaluate the stability of long-range structural correlations, the running integrals were computed independently for two consecutive 15 ns blocks (0–15 ns and 15–30 ns). The solid curves represent the structural ensemble averages across all 10 independent simulation replicas for each system, with the shaded envelopes showing the standard deviation across the full set of replicates.

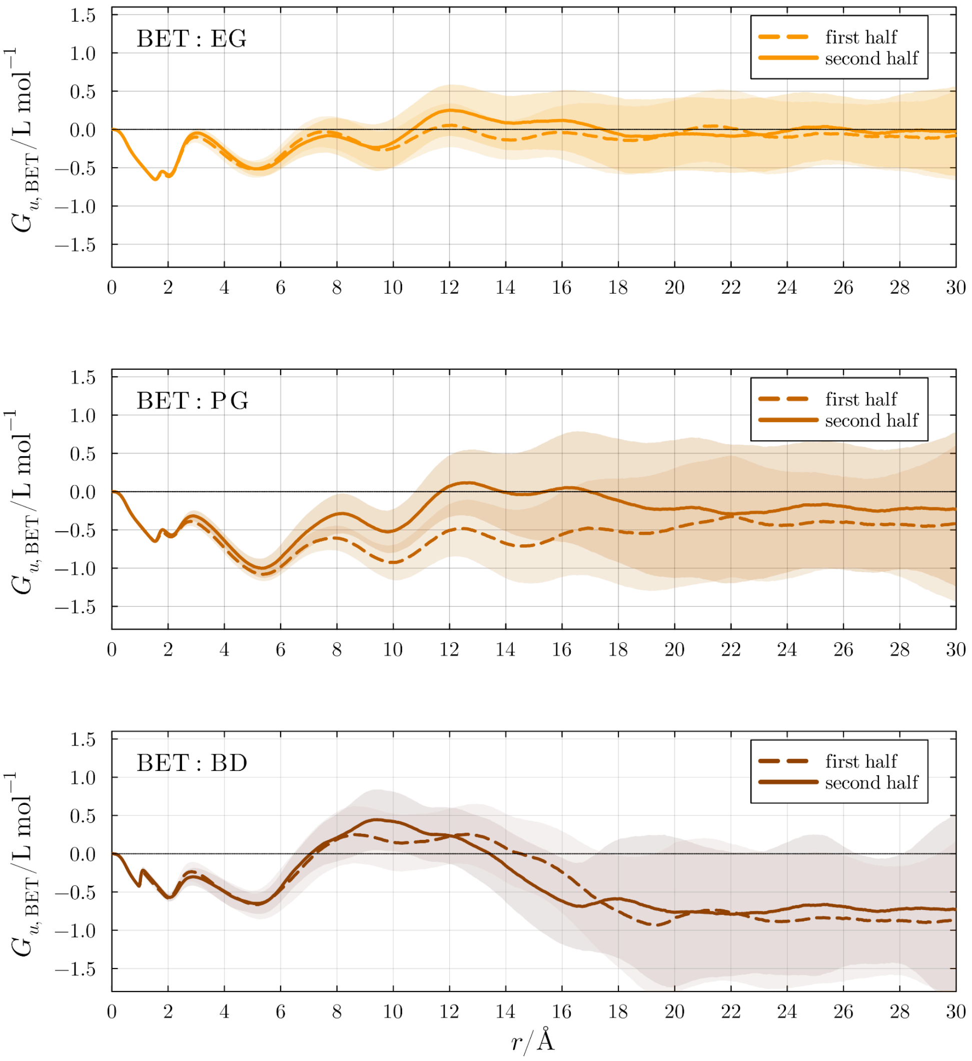

Figure S8. Long-range spatial convergence and block-decomposition analysis of the Kirkwood–Buff integrals for the hydrogen bond acceptor (HBA/betaine) species across the simulated deep eutectic solvents. To evaluate the stability of long-range structural correlations, the running integrals were computed independently for two consecutive 15 ns blocks (0–15 ns and 15–30 ns). The solid curves represent the structural ensemble averages across all 10 independent simulation replicas for each system, with the shaded envelopes showing the standard deviation across the full set of replicates.

Table S4. Block-decomposition and replica-averaged convergence analysis of Kirkwood–Buff integrals (G_{ij}) for the simulated deep eutectic solvents. To evaluate long-range structural stability, production trajectories were divided into two consecutive blocks of 15 ns each (0–15 ns and 15–30 ns). The reported values represent the converged macroscopic KB integrals (in L mol^{-1}) obtained from the stabilization plateau within the solute domain. Calculations were performed across all ten independent simulation replicas for each system, with errors expressed as the standard deviation encompassing the full ensemble of replicates.

System	KBI HBA - First 15 ns	KBI HBA - Last 15 ns	KBI HBD - First 15 ns	KBI HBD - Last 15 ns
**BET - EG**	-0.084 ± 0.110	-0.009 ± 0.131	-0.370 ± 0.266	-0.357 ± 0.260
**BET - PG**	-0.402 ± 0.340	-0.283 ± 0.320	-0.324 ± 0.300	-0.254 ± 0.326
**BET - BD**	-0.867 ± 0.540	-0.798 ± 0.568	-0.093 ± 0.304	-0.120 ± 0.370

References

1. Rodrigues, L. A. *et al.* Deep eutectic systems from betaine and polyols – Physicochemical and toxicological properties. *J. Mol. Liq.* **335**, 116201 (2021).
2. Goloviznina, K., Canongia Lopes, J. N., Costa Gomes, M. & Pádua, A. A. H. Transferable, Polarizable Force Field for Ionic Liquids. *J Chem Theory Comput* **15**, 5858–5871 (2019).
3. Canongia Lopes, J. N., Deschamps, J. & Pádua, A. A. H. Modeling ionic liquids using a systematic all-atom force field. *J. Phys. Chem. B* **108**, 2038–2047 (2004).
4. Canongia Lopes, J. N. & Pádua, A. A. H. CL&P: A generic and systematic force field for ionic liquids modeling. *Theor. Chem. Acc.* **131**, (2012).
5. Robertson, M. J., Tirado-Rives, J. & Jorgensen, W. L. Improved Peptide and Protein Torsional Energetics with the OPLSAA Force Field. *J Chem Theory Comput* **11**, 3499–3509 (2015).
6. Hanwell, M. D. *et al.* Avogadro: an advanced semantic chemical editor, visualization, and analysis platform. *J Cheminform* **4**, 17 (2012).
7. Tirado-Rives, J. & Jorgensen, W. L. Performance of B3LYP Density Functional Methods for a Large Set of Organic Molecules. *J Chem Theory Comput* **4**, 297–306 (2008).
8. Padua, A. *Fftool v1.0.0*. (Zenodo, 2015). doi:10.5281/ZENODO.18618.
9. Goloviznina, K., Gong, Z. & Padua, A. A. H. The CL &Pol polarizable force field for the simulation of ionic liquids and eutectic solvents. *Wiley Interdiscip. Rev. Comput. Mol. Sci.* **12**, (2022).
10. Lemkul, J. A., Huang, J., Roux, B. & MacKerell, A. D., Jr. An Empirical

Polarizable Force Field Based on the Classical Drude Oscillator Model: Development History and Recent Applications. *Chem Rev* **116**, 4983–5013 (2016).

11. Lamoureux, G. & Roux, B. Modeling induced polarization with classical Drude oscillators: Theory and molecular dynamics simulation algorithm. *J. Chem. Phys.* **119**, 3025–3039 (2003).
12. Lamoureux, G., MacKerell, A. D., Jr & Roux, B. A simple polarizable model of water based on classical Drude oscillators. *J. Chem. Phys.* **119**, 5185–5197 (2003).
13. Tang, K. T. & Toennies, J. P. An improved simple model for the van der Waals potential based on universal damping functions for the dispersion coefficients. *J. Chem. Phys.* **80**, 3726–3741 (1984).
